# Occupation-Based Life Expectancy: Actuarial Fairness in Determining Statutory Retirement Age

**DOI:** 10.3389/fsoc.2021.675618

**Published:** 2021-08-23

**Authors:** Dorly J.H. Deeg, Wouter De Tavernier, Sascha de Breij

**Affiliations:** ^1^ Department of Epidemiology and Data Science, Amsterdam University Medical Centers, Vrije Universiteit Amsterdam, Amsterdam, Netherlands; ^2^ Centre for Comparative Welfare Studies, Aalborg University, Aalborg, Denmark

**Keywords:** retirement age, occupation, longevity, health, longitudinal study

## Abstract

This study examines occupation-based differences in life expectancy and the extent to which health accounts for these differences. Twentyseven-year survival follow-up data were used from the Dutch population-based Longitudinal Aging Study Amsterdam (*n* = 2,531), initial ages 55–85 years. Occupation was based on longest-held job. Results show that the non-skilled general, technical and transport domains had an up to 3.5-year shorter life expectancy than the academic professions, accounting for the compositional characteristics age and gender. Statutory retirement age could be made to vary accordingly, by allowing a proportionally greater pension build-up in the shorter-lived domains. Health accounted for a substantial portion of the longevity difference, ranging from 20 to 66%, depending on the health indicator. Thus, health differences between occupational domains today can be used as a means to tailor retirement ages to individuals’ risks of longevity. These data provide a proof of principle for the development of an actuarially fair method to determine statutory retirement ages.

## Introduction

Actuarial fairness is considered a key aspect of a just pension system (Schokkaert and Van Parijs, 2003; De Tavernier, 2021) and can be understood as “equal treatment for equal risks” (Landes, 2015, p. 521). For a pension system to be actuarially fair, an individual’s contributions (plus interests) should equal the individual’s expected benefits. Expected benefits are directly linked to the expected duration of time during which they will be enjoyed, i.e., the individual’s life expectancy. It is well-known, however, that life expectancy is strongly socially stratified (Kunst and Mackenbach, 1994). Moreover, in European countries the longevity gap between lower and higher socio-economic positions has been shown to be increasing (Unger and Schulze, 2013; Östergren et al., 2017; Tanaka et al., 2019). Thus, equal treatment through a uniform statutory retirement age does not do justice to the socially highly unequally distributed life opportunities and is not actuarially fair (Unger and Schulze, 2013). Given a uniform statutory retirement age, Barnay (2007) showed that executives and the intermediate professions benefit much more from the pension system than manual workers. Likewise, several authors concluded that such pension systems generate a Matthew effect, redistributing means from the less well-off to the better-off (Myles, 2003; Schokkaert and Van Parijs, 2003; Queisser and Whitehouse, 2006; Simonovits, 2015). When contributions and benefits would be adapted to differences in life expectancy, redistribution towards an actuarially fair system could take place. This study proposes a mechanism through which this could be achieved.

Various policies affect actuarial fairness of pension systems. One way to make pension policies actuarially fair is through early retirement regulations. Some European countries offer early retirement possibilities for specific categories of workers in highly demanding jobs. In their overview, Natali et al. (2016) find a wide variety of classifications and interpretations of what constitutes arduous jobs across countries, and note that the concept is often not clearly delineated. This is the case for the new Finnish years-of-service pension (Finnish Centre for Pensions, 2015), for instance, where discretion is given to medical doctors to make decisions in individual cases. In other countries, such as Belgium and the Netherlands, social partners continue to disagree about which jobs fall in this category, illustrating the arbitrary nature of decisions about which jobs are particularly demanding.

This study aims to develop a more objective method to determine statutory retirement ages rooted in actuarial fairness by quantifying occupation-based life expectancies. Linking the resulting life expectancies to pension build-up would improve actuarial fairness of pension systems. Thus, workers in occupational domains with a shorter life expectancy should be facilitated to retire earlier by adjusting their pension build-up. It is important to note that by taking actuarial fairness as our point of departure, the question of whether inequalities in life expectancy are actually caused by occupational exposure differences is not relevant (indeed, selection into occupations might play an important role, leading to compositional differences, see Ravesteijn et al., 2018). The principle merely requires that pension policies are adjusted to observed differences in longevity: contributions and expected benefits should balance out–no matter what causes occupation-based differences in life expectancy.

Earlier evidence shows that numerous factors impact longevity, including physical and mental health related factors, social conditions, and heredity (e.g., Goldman et al., 2016; Iacob et al., 2016; Suemoto et al., 2017). Among these, social stratification indicators such as level of education and level of occupation play an important role. Many of these studies considered occupation as a proxy for socio-economic position. Indeed, in the United Kingdom occupational class has been used as the standard indicator of social class (Llena-Nozal et al., 2004). But also in studies outside the United Kingdom, occupation has been used as such, e.g., in Canada (Tjepkema et al., 2013), in Denmark (Brønnum-Hansen et al., 2020), and in other European countries (Tanaka et al., 2019). In these studies, the occupational classification represents a hierarchy of skill level, rather than actual occupations. It may be argued that social class is the main factor at work in occupation-related differences in health. Early studies by Moore and Hayward (1990) and Johnson and colleagues (1999), however, showed that important mortality differences between occupations exist that are not accounted for by social status, income, and education. A recent study by Leinonen et al. (2018) showed evidence of variation in sickness absence between four large industrial sectors despite the fact that these were examined within particular occupational classes. These studies support the unique contribution of occupation to health outcomes.

Evidence on the impact of employment characteristics other than job level on mortality comes from studies on working conditions. In a meta-analysis of 17 studies, Amiri and Behnezhad (2020) reported that in people with job strain, which is a combination of high demands and low control at work, the risk of mortality is 20% higher than in the reference group. Also physical demands have been shown to be associated with increased risk of mortality (Mikkola et al., 2019). In a recent study from our own group, people at age 55 who had physically strenuous jobs regarding repetitive movements and use of force had a 1.5–2.0 years shorter life expectancy than their counterparts with less strenuous jobs, and people who had jobs with a low variation in activities and low autonomy had a 1.1–2.5 years shorter life expectancy than their counterparts with jobs with high variation and autonomy (de Wind et al., 2020). Working conditions may be concentrated in specific occupations, but essentially cut across occupations. The findings from studies on working conditions are relevant for the management of older workers’ balance of work capacity and work demands, which is to be achieved mainly in the workplace and at the company level. For pension policy, however, it is useful to consider a higher aggregate level.

Studies addressing the impact on mortality of occupation per se are scarce, although they have a long tradition (Fox and Adelstein, 1978). Moore and Hayward (1990) showed that in the United States between 1966 and 1983, service workers had a 4.5-year shorter life expectancy from age 55 than professionals. In another US study, Johnson and colleagues (1999) found increasing risks of mortality across the occupational spectrum from the highly skilled occupations to less-skilled and generally more labor-intensive occupations. A more recent South Korean study of workers enrolled in a national insurance program showed that workers in elementary occupations had a twice as high mortality rate as professionals (Lee et al., 2016). A recent study in the United Kingdom was based on linkage of census data with 10-year follow-up registry mortality data and distinguished 60 occupations (Katikireddi et al., 2017). It showed that among men, the highest mortality rates were in elementary construction, housekeeping and factory workers, whereas health professionals had the lowest mortality. Among women, factory workers and garment trade workers had high, and teachers and business professionals had low mortality rates.

In this study, we will specifically address differences in longevity by broad occupational domains, as they closely correspond to industrial sectors. A sector is defined as a group of industries with the same main economic activity; the latter is indicated by the predominant type of occupation (Statistics Netherlands, 2021). Using sector as a study unit offers a number of advantages from a policy perspective. First, it suits the actuarial fairness argument as, in most countries, pension entitlements and contributions are linked to employment sector. Second, variations in pension policies can be decided by social partners in sector-specific collective bargaining, and occupational pension schemes often already are sector-specific (De Preter et al., 2012; Wiß 2015). Third, sector of employment is information that is easily accessible for governments and is readily available in many state registers. In Netherlands, the country in which our study is based, the focus on sector has direct applicability, as negotiations between unions of employees and employers are conducted sector-wise, and pension funds, the institutes that provide the work-related pension benefits, are organized within sectors. Hence, we formulate a first research question:

- *What are quantitative differences in longevity between occupational domains?*

We are aware of the issue that in developing occupation-based retirement ages, the calculation of occupation-based life expectancy involves mortality follow-up of individuals over time until enough individuals should have deceased in order to construct reliable mortality tables. This can only be done on historical data. However, occupations change over the years in terms of task use and demands (Romeu Gordo and Skirbekk, 2013; Cassidy, 2017), so that occupation-based life expectancies of earlier generations may not be generalized to those working in the same occupations today. To overcome this problem, we test to what extent the relation between occupational domain and life expectancy is accounted for by health in the historical data. We are not aware of earlier studies testing the role of health. However, if indeed health acts as an explanatory factor of this relationship, we can use health differences between domains today as a means to tailor statutory retirement ages to individuals’ risks of longevity, thereby making pension systems more actuarially fair. This leads to our second research question:

- *To what extent are health indicators explanatory factors in the association between occupational domain and longevity?*

## Materials and Methods

### Sample

Data are used from the 1992–93 baseline cycle of the Dutch population-based Longitudinal Aging Study Amsterdam (LASA), linked to vital status follow-up from municipal registries. LASA is a prospective study of cohorts based in three regions of the Netherlands that together form a representative sample for the Netherlands (Deeg et al., 2002; Hoogendijk et al., 2020). The baseline sample included 3,107 55–85-year-olds. Older ages and men were oversampled, so that each 5-year age group included about 250 male and 250 female participants. Among these participants, for 19 the vital status was unkown, 173 responded to a short version of the interview which did not include questions about current or past work, 80 missed one or more of the work questions, and 304 never had a job. Excluding these participants left a study sample size of 2,531.

### Dependent Variable

Vital status is traced periodically through the Municipal Personal Records database which covers all residents in the Netherlands. For the current study, mortality ascertainment up to December 31, 2019 was used, providing about 27 years of mortality follow-up. At the probing date, 84.0% if the study sample had died.

The dependent variable is operationally defined as the Realized Probability of Dying (RPD). The RPD is an individual measure of survival time relative to the total population, based on sex and age at baseline (Deeg et al., 1989). As such, the RPD belongs to the family of relative survival measures (Rutherford et al., 2012). We opt for this individual measure of survival rather than using commonly used group-based methods to predict survival time, such as Cox proportional hazards models, for several reasons. First, an individual measure is more accurate than group-based approaches. Second, it lends itself as the dependent variable in linear regression models, with the advantage that examination of explanatory factors (our second research question) is straightforward (Mood, 2010). Third, as the RPD is based on the age and sex of each individual participant, differences the in age-sex composition across occupational domains are accounted for. Differences in RPD can be transformed into differences in number of years of life expectancy.

Using life tables based on the total population for subsequent years (1992 through 2019) during the study period, the RPD compares for each individual of a specific age and sex this person’s survival probability with the overall survival probability of the Dutch population of that age and sex, from the starting month of the study through the years up to December 31, 2019. In formula:

$$\text{RPD}=\left({1\;-\text{ d}}_{1_{\;}}^{\left(\text{ai,s}\right)}\right)\ldots \left({1\;-\text{ d}}_{\text{n}}^{\left(\text{ai,s}\right)}\right)$$

where n is the total number of calendar years during which the participant is followed up to death or end-of-study, d_i_ is the probability of death according to the life table in calendar year i (i = 1 … n), a_i_ is the age in calendar year i, and s is the sex of the participant.

Possible values of the RPD lie between 0 and 1. These values introduce a rank order among all sample subjects. The reference population has a mean RPD of 0.50. If the RPD is greater than 0.50, this means that the subject has lived a relatively short time; if it is less than 0.50, the subject has lived a relatively long time after baseline. For example, the value of a man’s RPD is 0.80 if 80% of his age and sex peers in the total population are still alive at the time of his death. The name “realized probability of dying” comes from the notion that the individual has “realized” the probability of death when a certain percentage of the reference population is expected to be still alive. The actual amount of survival time needed to reach a particular RPD varies according to the age and sex of the individual at baseline, with older people needing less time to achieve a lower RPD than younger people, and men needing less time than women. For example, a man aged 65 years when first participating in LASA in 1993, who dies after 20 years in 2013, has an RPD of 0.39. By comparison, a woman aged 65 years in 1993 will have the same RPD of 0.39 when she dies after 24 years, in 2017.

For those participants still alive at the end of the study period (December 31, 2019), i.e. 16.0% of the study sample (*n* = 406), a value of the Realized Probability of Dying is imputed. The RPD for these participants is estimated by assuming that their remaining survival time corresponds to the median population survival time from end-of-follow-up onward. This amounts to multiplying the probability of reaching their age at the end of 2019 by 0.5. For example, a woman aged 65 when examined in 1993, reaches the age of 91 in 2019 with probability 0.30. If she is still alive at the end of 2019, her imputed RPD is 0.15, implying that it is expected that she will die when only 15% of her 1993 cohort is still alive. This approach is derived from standard actuarial methods.

If the study sample’s RPD shows a uniform distribution, the survival distribution of the sample represents that of the total population. In this case, the logit of the RPD (LRPD = log(RPD/(1-RPD))) approaches a normal distribution with mean 0, and can be used as the dependent variable in analysis of variance and linear regression analysis. In our study sample, the mean (standard deviation) of the RPD and the LRPD are 0.50 (0.28) and 0.05 (1.66), respectively, and thus its survival is very close to that of the total population.

### Independent Variables

#### Occupational Domains

For baseline participants who currently did paid work, their precise job description was recorded. For those who had done paid work in the past, the job description of their longest-held job was recorded. For those participants whose current job was not the longest-held job, data on their longest-held job were used, because workers may have moved to less strenuous jobs and for them, the association of occupation with longevity may be underestimated (Moore and Hayward, 1990). Based on the job characteristics domain (e.g., agricultural, care, teaching), required skill level (i.e., elementary through scientific, based on required education, training period, and work experience), and tasks (e.g., cattle breeding, nursing, instructing), jobs were classified into 43 occupational categories according to the Netherlands Standard Classification of Occupations 1992 (Statistics Netherlands, 2001; Rijs et al., 2014). These categories were condensed into 13 broad occupational domains by collapsing the skill levels within one domain. For the current study, seven of the largest domains were selected: the non-skilled general, technical, transport, administrative/commercial, care, agriculture, and pedagogical (teaching) domains. The technical domain includes jobs such as construction, machine work, electro-technical maintenance. The administrative/commercial domain includes jobs such as book keeper, buying clerk, hotel manager. The care domain includes (para)medical and social care and services jobs. Together, these seven domains constitute 91.7% of the sample. Domains that had less than 90 cases were grouped and served as the reference category for comparison of survival time. This category includes the following domains: natural science, juridical/security, cultural/linguistic, social science, and management. All had a survival time longer than average (Supplementary Table A). The mean (standard deviation) of the LRPD of the reference group was −0.21 (1.61).

#### Health

In order to facilitate implementation in the practice of pension insurance, we selected five health measures that are commonly retrievable from register data for initial analyses, i.e., sickness days, number of medications, hospital admission, outpatient visits, and general practitioner contact. For sensitivity analyses, we selected seven other health measures that have been proven to be a “best” predictor set for longevity (Suemoto et al., 2017).

*Sickness days.* These were self-reported as the number of days during the past month that participants had been ill to such extent that they had to stay in bed, with response categories 1) no days, 2) 1–3 days, 3) 4–7 days, 4) more than 1 week but less than a month, 5) all month.

*Number of medications*. The medications which the participants were using were recorded by the interviewer by inspecting the medicine containers. The total number of medications used was included in the analyses.

*Hospital admission* was self-reported. In order to avoid recall bias, hospital admission was not asked longer back than 6 months.

*Outpatient visits*. Contact with a medical specialist or psychiatrist was self-reported and also pertained to the past 6 months.

*General practice contact*. Contact with the participant’s general practitioner was self-reported and pertained to the past 6 months.

*Four chronic conditions*, i.e., obstructive lung disease, cardiovascular disease, diabetes, and cancer, were self-reported. Comparison with general practitioner records showed a satisfactory agreement (Kriegsman et al., 1996).

*Disability* was self-reported using the Global Activity Limitation Index, which asks about activity limitation that has lasted at least 3 months. It is coded as 1) no limitations, 2) mild limitations, 3) severe limitations (Van Oyen et al., 2006).

*Self-rated health* was measured using the single, widely used question “How is your health in general?,” with codes from 1) very good, to 5) poor.

*Cognitive impairment* was ascertained using the Dutch translation of the MiniMental State Exam (MMSE, Folstein et al., 1975). On 23 questions and tasks, respondents received one or more points when they gave the correct answer or performed the task correctly. Scores range from (0) all answers incorrect, to (30) unimpaired.

#### Covariates

Other independent variables, for descriptive purposes, include socio-demographic characteristics: age, gender, education in years, and occupational skill level. The latter variable was coded from 1) elementary to 5) scientific. Work status was included in the analyses, because evidence shows that health tends to improve after retirement (Eibich, 2015), and that before retirement age, people who currently do paid work are in better health than people who do not do paid work (Scharn et al., 2019). Work status was defined using two dummy variables: doing paid work versus not doing paid work, and being fully retired versus not fully retired.

### Statistical Analysis

The socio-demographic and health characteristics of the seven occupational domains were compared using the chi-square test for dichotomous variables, and ANOVA’s F-test for continuous variables. Likewise, the distribution of the LRPD across the domains was examined using ANOVA’s F-test. To provide an illustration of this distribution, the domain-specific remaining life expectancies from age 65 were calculated based on the mean RPD of each domain, and shown in a figure. Subsequently, a series of linear regression analyses was performed to test survival time differences. The first model included only the domain dummy variables and age; in a second model work status was added. A next series of linear regression models included in addition one health variable at a time, and examined to what extent this health variable accounted for the association between domain membership and survival time, by comparing the coefficient of each domain dummy in the models without and with the health variable and calculating the percentage decline in this coefficient. In a final model, all five health variables were included and again, the percentage decline in the domain dummy coefficients was calculated. In a sensitivity analysis, the domain coefficients in the models without and with the ‘best’ predictor set of seven health variables were compared. As 14.6% of the sample had missing values on one or more variables, 12.8% of which was due to medication use only, multiple imputation was applied. Fifteen imputations were performed. If a health variable showed a non-linear association, its quadratic term was tested in the case of a continuous variable, or dummies were created.

## Results

### Descriptives

At baseline, 333 participants currently had a paid job, whereas 2,198 had no current job but had held a job earlier in life. 76 participants had moved from their longest-held job to their current job, 71 of which currently worked in a different occupational domain.

Table 1 shows the distribution of socio-demographic characteristics across the domains. The percentage of males is highest in the transport domain (92.9%), followed by the agricultural and technical domains (79.4 and 77.5%, respectively), and lowest in the care domain (12.5%). The average level of education is highest in the teaching domain (13.6 years) and lowest in the non-skilled general domain (6.8 years). Likewise, average skill levels are highest in the teaching and “other” domains (4.3 and 4.0) and lowest in the elementary domain (1.0), but among the other five domains, there are no clear differences (circa 2.6). Note, that the standard deviation of years of education is relatively high in the care domain, reflecting that this domain includes substantial numbers of both lower and higher educated workers. The highest percentages of currently paid workers are observed in the agriculture and teaching domains (21.1 and 17.1%, respectively). In the non-skilled general, care and administrative domains, the highest percentages of people without paid work younger than 65 years and not retired are observed (21.9, 21.3, and 21.2%, respectively). The highest percentages of (early) retirees are found in the transport and technical domains (76.8 and 74.7%, respectively). Differences between domains regarding all socio-demographic characteristics are statistically significant at *p* < 0.001, indicating cross-domain heterogeneity.

**TABLE 1:** Socio-demographic characteristics of the occupational domains selected. Source: Longitudinal Aging Study Amsterdam, 1992–93 (*n* = 2,531).

	N (%)	Gender^a^ (%)		Education in years^b^	Skill level^c^	Work status^d^ (%)			LRPD^e^
		Male	Female	M (sd)	M (sd)	Paid work	Age <65, no paid work, not retired	(Early) retired	M (sd)
General	224 (8.9)	29.9	70.1	6.8 (1.9)	1.0 (0.1)	8.0	21.9	70.1	0.17 (1.68)
Technical	679 (26.8)	77.5	22.5	8.6 (2.8)	2.6 (0.7)	11.5	13.8	74.7	0.15 (1.66)
Transport	99 (3.9)	92.9	7.1	7.4 (2.3)	2.5 (0.5)	10.1	13.1	76.8	0.26 (1.69)
Administrative	618 (24.4)	43.6	56.4	9.4 (2.8)	2.7 (0.6)	14.3	21.2	64.6	0.02 (1.64)
Care	397 (15.7)	12.5	87.5	8.3 (3.5)	2.6 (0.8)	11.5	21.3	66.2	−0.04 (1.60)
Agriculture	194 (7.7)	79.4	20.6	8.1 (2.3)	2.7 (0.5)	21.1	8.2	70.6	−0.20 (1.65)
Teaching	107 (4.2)	46.8	53.2	13.6 (3.1)	4.3 (0.5)	17.1	10.8	72.1	−0.27 (1.71)
Others	211 (8.3)	52.3	47.7	9.5 (4.1)	4.0 (0.8)	12.6	14.6	72.8	−0.26 (1.61)

a Gender differences between the domains are significant at *p* < 0.001.

b Educational differences between the domains are significant at *p* < 0.001.

c Occupational skill level (range 1 = elementary … 5 = scientific) differences between the domains are significant at *p* < 0.001.

d Work status differences between the domains are significant at *p* < 0.001.

e Logit of the Realized Probability of Dying; differences between the domains are significant at *p* = 0.003.

The distribution of the five key health indicators across the occupational domains is shown in Table 2. Although health differences between the selected domains and the “other” domain are apparent, only few differences reach statistical significance at *p* < 0.05. Considering only these differences, it is seen that workers in the technical domain have relatively many and in the agriculture domain have relatively few sick days; workers in the non-skilled general domain use relatively many medications and are relatively often admitted to a hospital; and workers in the technical domain have relatively few family physician contacts.

**TABLE 2:** Key health characteristics of the occupational domains selected. Source: Longitudinal Aging Study Amsterdam, 1992–93 (*n* = 2,531).

	≥1 sick days past month (%)	No. of current medications (M, sd)	Hospital admission^a^ (%)	Outpatient visit^a^ (%)	Family physician contact^a^ (%)
General	8.1	2.0 (1.8)^d^	14.0^c^	48.3	74.6
Technical	10.3^c^	1.8 (1.8)	11.4	50.4	68.0^c^
Transport	8.1	1.7 (1.8)	11.9	57.5^b^	74.6
Administrative	9.4	1.6 (1.8)	8.1	49.6	73.6
Care	6.1^b^	1.7 (1.8)	9.2	44.7^b^	69.6
Agriculture	3.1^d^	1.5 (1.6)	8.4	42.3^b^	73.1
Teaching	6.5	1.5 (1.7)	10.4	50.5	70.0
Others	8.1	1.6 (1.8)	8.1	51.0	75.2

a In the past 6 months.

Significance of difference between each domain and the “others” domain:

b *p* < 0.10;

c *p* < 0.05;

d *p* < 0.01.

### Association of Occupational Domain With Life Expectancy

**FIGURE 1:**
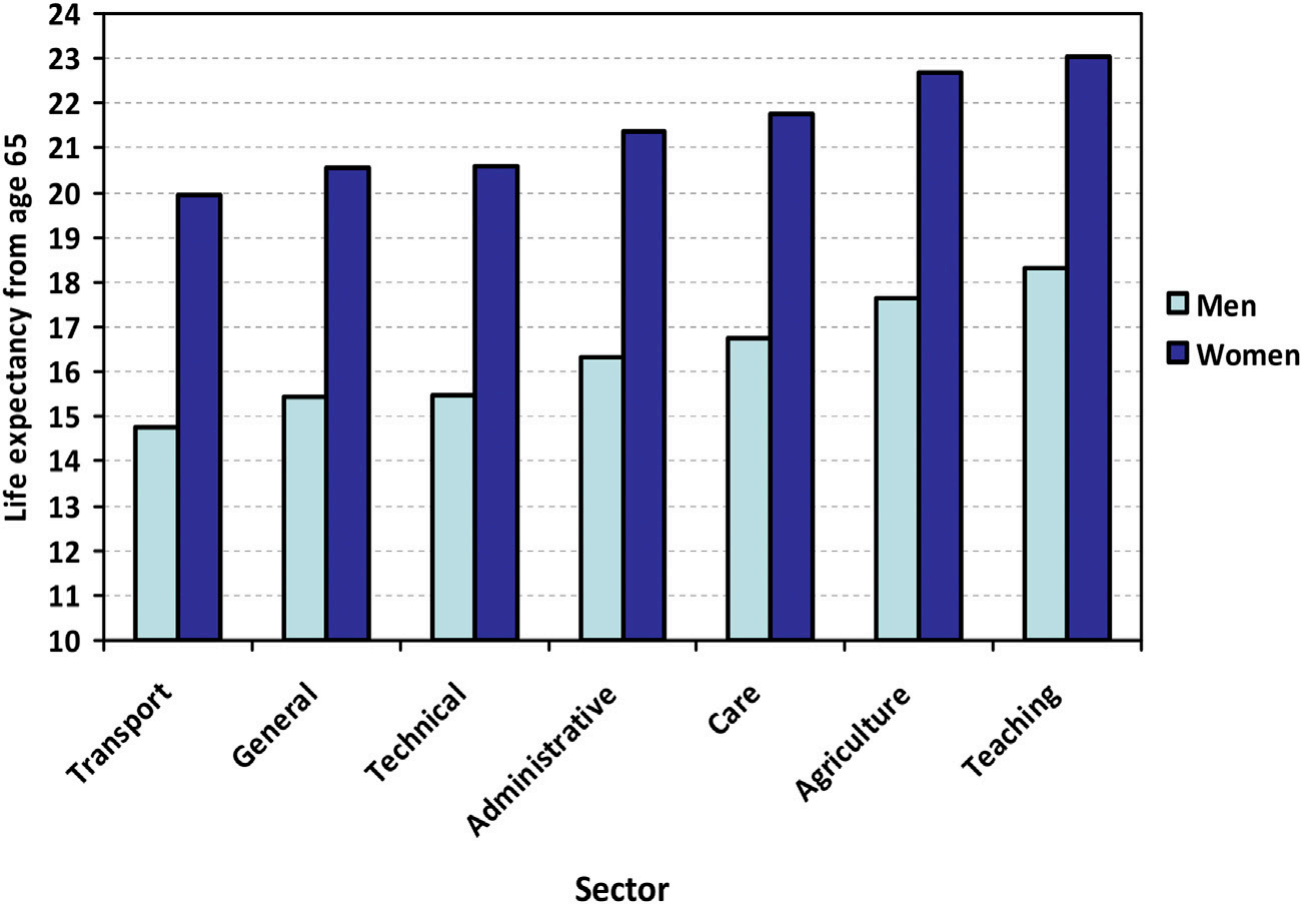
Life expectancy by occupational domain for men (light colors) and women (dark colors) at age 65. Note: The data are based on analysis of domain specific relative survival using the full sample, the results of which are compared to the population-based sex-specific survival curves at age 65 in order to obtain life expectancies (Supplementary Figure A). Source: Longitudinal Aging Study Amsterdam, 1992–93 to 2019.

The Logit of the Realized Probability of Dying (LRPD) ranges from +0.26 for the transport domain to −0.27 for the teaching domain (overall cross-domain difference: *p* = 0.003, Table 1). Figure 1 shows the LRPD-derived remaining life expectancies for men and women at age 65. The transport domain is characterized by the shortest life expectancy, i.e., 14.7 years for men and 20.0 years for women. The teaching domain is characterized by the longest life expectancy, i.e., 18.3 years for men and 23.1 years for women. For men, the difference amounts to 3.6 years; for women, this is 3.1 years. In-between are, in ascending order, the non-skilled general, technical, administrative, care, and agriculture domains. The population median of the survival time at age 65 is 16.5 years for men and 21.5 years for women. Thus, on the lowest end of the spectrum, the transport domain’s life expectancy is 1.8 years below the population median for men and 1.5 years for women. On the other end of the spectrum, the survival time advantage of the teaching domain is 1.8 years for men and 1.6 years for women. These differences would imply that male workers in the transport domain would be allowed for example a 1 + 1.8/16.5 = 1.1 greater pension build-up during their working years, accompanied by a proportionally earlier statutory retirement age than male workers in a domain with a median life expectancy.

A further test of these differences using linear regression analysis (model 1 in Table 3) yields statistically significantly higher LRPDs for, in descending order, the transport, the non-skilled general, the technical, and the administrative domain as compared to the other, non-defined domains (Bs ranging from 0.503 to 0.264; *p* < 0.05). Adding work status to the regression model somewhat decreases the regression coefficients, such that the coefficient for the administrative section no longer reaches statistical significance (model 2 in Table 3).

**TABLE 3:** Linear regression models of LRPD on occupational domain^1^, adjusted for age (model 1), and adjusted for age and work status (model 2). Source: Longitudinal Aging Study Amsterdam (*N* = 2,531).

	Model 1			Model 2		
	Regression coefficient B	Confidence interval	Significance (*p*-value)	Regression coefficient B	Confidence interval	Significance (*p*-value)
General	0.422	0.112; 0.732	0.008	0.388 (0.158)	0.078; 0.698	0.014
Technical	0.405	0.150; 0.660	0.002	0.395 (0.130)	0.140; 0.650	0.002
Transport	0.503	0.111; 0.895	0.012	0.499 (0.200)	0.107; 0.891	0.013
Administrative	0.264	0.007; 0.521	0.045	0.243 (0.132)	−0.014; 0.500	0.066
Care	0.204	−0.070; 0.478	0.146	0.175 (0.141)	−0.101; 0.451	0.213
Agriculture	0.065	−0.254; 0.384	0.690	0.060 (0.163)	−0.259; 0.379	0.712
Teaching	−0.022	−0.404; 0.360	0.910	−0.024 (0.195)	−0.406; 0.358	0.903
Paid work	-	-	-	−0.282 (0.114)	−0.505; −0.059	0.014
(Early) retired	-	-	-	−0.275 (0.122)	−0.514; −0.036	0.024

a Each domain is compared to the “others” domain.

### The Role of Health

To test the role of health, first, in five separate regression analyses, one for each health variable, the occupational domain coefficients are compared between non-health-adjusted and health-adjusted models (Supplementary Table B). It is observed that number of sick days, number of medications, hospital admission, and outpatient visits are strongly associated with survival time, but family physician contact is not. Of all health variables, the number of medications explains the largest portion, ranging from 16.2% for the non-skilled general domain to 9.1% for the administrative domain. Contact with the family physician does not contribute any explanatory value.

The final model including all five health variables yields similar decreases in the B-coefficients of the non-skilled general, transport, technical, and administrative domains, ranging between 19.0 and 20.9% (Table 4).

**TABLE 4:** Linear regression models of LRPD on occupational domain^1^, adjusted for age and work status (model 1), and additionally adjusted for five health variables (models 2). Imputed data (*n* = 2,531)^2^. Source: Longitudinal Aging Study Amsterdam, 1992–93 to 2019.

	Model 1			Model 2		
	Regression coefficient B	Confidence interval	Significance (*p*-value)	Regression coefficient B	Confidence interval	Significance (*p*-value)
General	0.344	0.032; 0.656	0.030	0.273	−0.029; 0.575	0.076
Technical	0.383	0.128; 0.638	0.003	0.308	0.061; 0.555	0.015
Transport	0.484	0.092; 0.876	0.016	0.392	0.010; 0.774	0.044
Administrative	0.244	−0.015; 0.503	0.090	0.193	−0.058; 0.444	0.130
Care	0.155	−0.121; 0.431	0.272	0.122	−0.147; 0.391	0.372
Agriculture	0.047	−0.274; 0.368	0.773	0.113	−0.199; 0.425	0.475
Teaching	−0.038	−0.420; 0.344	0.845	−0.040	−0.410; 0.330	0.832
≤1 sick days < all month^a^	-	-	-	0.187	−0.044; 0.418	0.111
Sick all month^a^	-	-	-	1.960	0.900; 3.020	<0.001
Medications^b^	-	-	-	0.038	0.030; 0.046	<0.001
Hospital admission	-	-	-	0.249	0.024; 0.474	0.031
Outpatient	-	-	-	0.140	0.001; 0.279	0.050
Family physician	-	-	-	−0.181	−0.328; −0.034	0.016

^1^Each domain is compared to the “others” domain.

^2^Pooled data based on 15 imputations.

^a^Two dummy variables, reference category is 0 sick days.

^b^Quadratic term.

### Sensitivity Analysis

The ‘best’ predictor set of seven health measures accounts for a substantially larger portion of the association between occupational domains and longevity (Supplementary Table C). The greatest decrease in the B-coefficient is observed for the non-skilled general domain, amounting to 66.5%. For the technical and transport domains, the percentage decrease is 48.1 and 41.9%, respectively. For the administrative domain, the portion accounted for is 20.2%, and thus similar to that using the initial five health measures.

## Discussion

In this study, we provided evidence regarding the extent of quantitative differences in longevity between occupational domains. As a proof of principle for the determination of actuarially fair statutory retirement ages, we indeed found domain differences, in that the non-skilled general, technical, and transport domains had significantly shorter survival times than the domains with academic professions. These findings correspond to the scarce literature that links occupational domain to mortality (e.g., Moore and Hayward, 1990; Johnson et al., 1999, Katikireddi et al., 2017, Lee et al., 2016). Furthermore, we found for four of our five health indicators which are presumably retrievable from registries, that they explained a substantial portion of the association of domain with longevity. The joint contribution of these health variables was about 20% for the domains with the shortest life expectancy. The contribution of a “best” predictor set of health indicators ranged from 66.5 to 20.2%, with the largest percentage in the domain with the shortest life expectancy. These findings suggest that health differentiates among occupational domains in a similar way as life expectancy.

Comparison of the differences in life expectancy found in our study with the pertinent scientific literature is not straightforward. In reports from the few studies addressing the association of occupation or occupational domain with mortality, the findings are commonly expressed as risk ratios or percentages excess mortality. Therefore, differences in life expectancy expressed in years, such as our study reports, are not directly comparable. The only study reporting between-domain differences in life expectancy in years, to our knowledge, showed a maximum difference of 4.5 years at age 55 (Moore and Hayward, 1990). Considering the mean life expectancy at age 55 is larger than at age 65, this difference is comparable to the one we found at age 65. Our earlier study on work conditions and life expectancy at age 55 showed a maximum difference between unfavorable and favorable work conditions of 2.6 years for men and 2.3 for women (de Wind et al., 2020). The latter are smaller than the domain differences found in the current study. A German study in 16–65-year-olds of the effect of working conditions on self-rated health found a 16-months “ageing effect” of high physical strain, and a 6-months effect of low control (Ravesteijn et al., 2018). Although the use of a different health measure and different occupational characteristics do not allow a close comparison with our own study, these effects are also smaller than the domain-effects on survival time in our current study. Thus, occupational domain is certainly a relevant criterion to differentiate shorter from longer life expectancies.

It may be argued that health behaviors are a major factor at work in occupation-related differences in health, because unhealthy behaviors are shared within occupations (Fox and Adelstein, 1978; Johnson et al., 1999). The implication would be that differences in longevity by occupational domain arise from behavior that is independent from employment and thus should not be accounted for in pension policy. One study of workers in the construction sector compared the variance explained in work ability by health conditions and health behaviors on the one hand and work-related factors on the other hand (Alavinia et al., 2007). Health behaviors, including obesity, physical activity, and smoking, explained less than 1% over and above age and occupational status. In contrast, the explanatory value of the work-related factors, including both physical and psychosocial demands, was 22%. Several other studies on work ability adjusted for health behaviors and provide evidence that occupational factors impact health outcomes independently of health behavior (Lund and Csonka, 2003; Andersen et al., 2016; Schram et al., 2021). In an extra analysis of our own dataset, we have added the lifestyle factors smoking, heavy alcohol consumption, obesity, and minutes spent on physical activity (walking, doing a sport) as confounders to our basic analytic model (i.e., model 2 in Table 3). Interestingly, these factors acted as suppressors, i.e., the regression coefficients for the general, technical and transport domains became stronger. This was due to the fact that the domain “other” included substantially more current smokers and heavy alcohol consumers than the specified domains, while this category had a relatively long survival time. In our study, therefore, unhealthy behaviour does not explain the association between domains and longevity. These findings once again support the relevance of occupational domain for pension policy.

From the identification of health indicators as explanatory factors, two kinds of implications can be noted. One is, in line with our second research question, to differentiate between occupational domains in determining pensionable ages. The other one is to improve health in the workplace. Numerous observational studies on work and health have noted the latter implication. However, health intervention studies in the workplace so far do not show very promising results, i.e. effect sizes are small, if any (e.g., Hazelzet et al., 2019; Söderbacka et al., 2020). Until effective interventions are designed, differentiation of pension policies based on health differences between occupational domains is recommendable to reach greater actuarial fairness.

### Strengths and Limitations

We emphasize that our findings should be considered as preliminary. Nevertheless, we can already state some strengths and limitations. The sample used is representative for the older Dutch population in 1992–93. Our analyses use occupational domain as the basis, because domains closely correspond to industrial sectors, and in the Netherlands pension policies are negotiated by social partners in sector-specific collective bargaining and occupational pension funds are organized by sector. Thus, implementation of our model is facilitated. The measure of survival time used is sensitive to inter-individual differences, because it is based on age and gender, and is prospective. Furthermore, our exposure variable was longest-held job rather than current job, so that health selection through transition to less strenuous jobs was precluded (Moore and Hayward, 1990).

In addition to these strengths, there are some weaknesses. First, the sample is relatively small when it comes to studying occupational domains more comprehensively. We selected the seven largest domains, based on a rule of thumb that their sample size is not smaller than 90. This makes it difficult to compare our findings to studies that had much larger sample sizes available and were able to make finer distinctions (e.g., Katikireddi et al., 2017). Also, the reference category consisted of the non-selected domains, in which a variety of smaller domains was collapsed, although they generally consisted of professional occupations, with concomitant greater survival times. With a larger sample size, we might have been able to observe clearer survival differences between domains. Furthermore, as there are gender differences in lifetime careers, even within a domain (Cambois et al., 2017; Riekhoff and Järnefelt 2017; Amiri and Behnezhad, 2020) and women tend to live longer than men, gender-specific analyses would have been preferable, but were not possible due to the relatively small sample size. However, as the survival measure LRPD is based on sex and age, gender differences in longevity were accounted for.

A second issue is that our sample has a broad age range (55–85 years). This implies that survival effects were examined of working in occupational domains, the exposure to which may have taken place decades earlier. Possibly, the effects of this exposure have weakened over the years. Regardless, we still find substantial survival time differences between domains. The long-lasting effect of working conditions on health is supported by studies of post-retirement health that showed that the health effects of poor working conditions lasted as long as 15 years post-retirement (Gueorguieva et al., 2009; de Breij et al., 2019).

A third limitation is that we were not able to incorporate the fact that individuals do not remain in the same occupation throughout their full careers (Kreiner et al., 2018). However, we did include the job that the participants had held during the longest time. Moreover, for participants currently working, their longest-held job was used if their current job was not the same as their longest-held job. Unfortunately, we did not have information on the duration of working in each occupational domain. In future research, using such information, work histories may be constructed for those participants who changed domain during their working career. Then, “weights” could be assigned to career years depending on domain. For example, 1 year working in non-skilled general occupations could be counted as 1.1 years in pension build-up.

A fourth issue may be that we initially selected health measures that could be retrieved relatively easily from registers available to pension funds. Although four of the five measures proved to be strongly associated with survival time, together they explained only 20%, a substantial yet relatively small portion of the association of occupational domain and survival time. In a sensitivity analysis, more direct health measures that have been shown to be strong and consistent predictors of longevity (Suemoto et al., 2017), including diseases and impairments, explained much greater portions, with a maximum of 66% for non-skilled general occupations. Although these measures are less likely to be available in registers, they confirm that for the longevity of non-skilled and skilled manual occupational domains, health is a strong explanatory factor.

Fifth, it may be argued that we included only a few covariates. Again, this choice was motivated by the likely availability of covariates in registers available to pension funds. We would like to stress, meanwhile, that we were primarily interested in longevity differences between occupational domains per se, because of their applicability in practice. Thus, we did not pursue a study of the unique predictive ability of domain for survival time given other individual and work-related characteristics.

As a final point, it must be acknowledged that working conditions within an occupational domain may vary substantially, which may result in substantial differences in life expectancy. Translated to industrial sectors, thus, unfairness within sectors may remain when applying sector-based pension rules. This may have implications for public acceptance of such rules, and may reduce predictability of pension benefits and timing.

To apply research findings in practice, the uncertainty that surrounds estimates and that is inherent in survey research should be minimized. As a further step, therefore, we recommend that the same research questions are addressed using national data from countries that have long-standing population-wide registry data available. Using such data would solve the sample size related limitations of our study. Due to somewhat different occupational distributions and prevalence of part-time work, however, the effects of having a certain occupation on health and life expectancy might vary across countries and time periods. Hence, the comparison would also act as a “robustness check” of the proposed mechanism, to see if its performance is dependent on certain circumstances. For the current study, however, we started with Dutch data with a long survival follow-up in order to provide a proof of principle.

## Conclusion

In this study, we aimed to provide a proof of principle of a mechanism to reach greater actuarial fairness by linking retirement ages to occupation-based life expectancies. We showed that the non-skilled general, technical, and transport domains have a shorter life expectancy than the professional domains, amounting to 3.5 years for a man, and 3.1 years for a woman aged 65 years. Statutory retirement age could be made to vary accordingly, by allowing a proportionally greater pension build-up in the shorter-lived domains. Also, we were able to show that health accounted for a substantial portion of the association between occupational domain and longevity. Thus, health differences between domains today can be used as a means to tailor retirement ages to individuals’ risks of longevity. This method provides a basis for pension policies of greater actuarial fairness, by linking pension build-up and statutory retirement ages to occupation-based life expectancies, instead of holding on to a one-size-fits-all statutory retirement age.

## Data Availability

A publicly available dataset was analyzed in this study. This dataset can be found here: https://lasa-vu.nl/en/request-data/. Data from the Longitudinal Aging Study Amsterdam are available for use for specific research questions provided that an agreement is made up.
