# Supplementary material for: Occupation-Based Life Expectancy: Actuarial Fairness in Determining Statutory Retirement Age
Source: Front Sociol. 2021 Aug 23;6:675618. doi: 10.3389/fsoc.2021.675618 (PMC8419329; doi:10.3389/fsoc.2021.675618)
Supplement: Supplementary file 1 [file DataSheet1.docx]

Supplement to

**Occupation-Based Life Expectancy: Actuarial Fairness in Determining Statutory Retirement Age**

Table A. Socio-demographic characteristics of the non-selected occupational domains. Source: Longitudinal Aging Study Amsterdam, 1992-93 (n=435)

Table B. Linear regression models of LRPD on occupational domain, adjusted for age and work status (model 1), and additionally adjusted for single health variables (models 2). Imputed data (n=2,531). Source: Longitudinal Aging Study Amsterdam, 1992-93 to 2019.

Table C. Linear regression models of LRPD on occupational domain, adjusted for age and work status (model 1), and additionally adjusted for ‘best’ health variables (models 2). Imputed data (n=2,531). Source: Longitudinal Aging Study Amsterdam, 1992-93 to 2019.

Figure A. Sex-specific survival curves at age 65, derived from the Realized Probability of Dying. The horizontal dashed line indicates the median RPD. The points where this line crosses the survival curves indicate the average life expectancies of men and women at age 65; these life expectancies can be read on the x-axis by following the vertical dashed lines and subtracting 1993.

Table A. Socio-demographic characteristics of the non-selected occupational domains. Source: Longitudinal Aging Study Amsterdam, 1992-93 (n=211)

|  |  | Gender (%) | | Education in years | Skill level | Work status (%) | | | LRPD^1^ |
| --- | --- | --- | --- | --- | --- | --- | --- | --- | --- |
|  | N (%) | Male | Female | M (sd) | M (sd) | Paid work | Age < 65, no paid work, not retired | (Early) retired | M (sd) |
| Natural science | 31 (14.7) | 61.3 | 38.7 | 12.1 (4.3) | 3.9 (0.8) | 0.0 | 3.2 | 96.8 | -0.28 (1.39) |
| Juridical/security | 63 (29.9) | 93.7 | 6.3 | 12.0 (3.8) | 3.6 (1.0) | 12.7 | 6.3 | 81.0 | -0.19 (1.29) |
| Cultural/linguistic | 35 (16.6) | 68.6 | 31.4 | 11.3 (3.7) | 3.7 (0.5) | 28.6 | 11.4 | 60.0 | -0.33 (1.93) |
| Social Science | 45 (21.3) | 53.3 | 46.7 | 13.6 (3.7) | 4.1 (0.8) | 26.7 | 6.7 | 66.7 | -0.14 (1.39) |
| Management | 37 (17.5) | 91.9 | 8.1 | 12.7 (4.0) | 4.7 (0.5) | 13.5 | 8.1 | 78.4 | -0.49 (2.17) |
| Total | 211 (100.0) | 75.8 | 24.2 | 12.4 (3.9) | 4.0 (0.8) | 16.6 | 7.1 | 76.3 | -0.26 (1.61) |

^1^ Logit of the Realized Probability of Dying

Table B. Linear regression models of LRPD on occupational domain^1^, adjusted for age and work status (model 1), and additionally adjusted for single health variables (models 2). Imputed data (n=2,531)^2^

|  | Model 1 | | | Model 2 | | | |
| --- | --- | --- | --- | --- | --- | --- | --- |
|  | Regression  coefficient B | Confidence  Interval | Significance  (p-value) | | Regression  coefficient B | Confidence  Interval | Significance  (p-value) |
| *Sick days past month** | | | | | | | |
| General | 0.344 | 0.032; 0.656 | 0.030 | | 0.336 | 0.026; 0.646 | 0.034 |
| Technical | 0.383 | 0.128; 0.638 | 0.003 | | 0.366 | 0.111; 0.621 | 0.005 |
| Transport | 0.484 | 0.092; 0.876 | 0.016 | | 0.462 | 0.070; 0.854 | 0.021 |
| Administrative | 0.224 | -0.035; 0.483 | 0.090 | | 0.211 | -0.046; 0.468 | 0.109 |
| Care | 0.115 | -0.161; 0.391 | 0.272 | | 0.163 | -0.111; 0.437 | 0.246 |
| Agriculture | 0.047 | -0.274; 0.368 | 0.773 | | 0.052 | -0.267; 0.371 | 0.750 |
| Teaching | -0.038 | -0.420; 0.344 | 0.845 | | -0.033 | -0.413; 0.347 | 0.865 |
| 1<= sick days < all month |  |  |  | | 0.314 | 0.081; 0.547 | 0.008 |
| Sick all month |  |  |  | | 2.209 | 1.139; 3.279 | <0.001 |
| *Number of medications*** | | | | | | | |
| General | 0.388 | 0.078; 0.698 | 0.014 | | 0.325 | 0.023; 0.627 | 0.035 |
| Technical | 0.395 | 0.140; 0.650 | 0.002 | | 0.349 | 0.102; 0.596 | 0.006 |
| Transport | 0.499 | 0.107; 0.891 | 0.013 | | 0.442 | 0.058; 0.826 | 0.024 |
| Administrative | 0.243 | -0.016; 0.502 | 0.065 | | 0.221 | -0.030; 0.472 | 0.085 |
| Care | 0.175 | -0.101; 0.451 | 0.213 | | 0.139 | -0.130; 0.408 | 0.311 |
| Agriculture | 0.060 | -0.259; 0.379 | 0.712 | | 0.123 | -0.189; 0.435 | 0.441 |
| Teaching | -0.024 | -0.406; 0.358 | 0.903 | | -0.014 | -0.386; 0.358 | 0.941 |
| Medications |  |  |  | | 0.041 | 0.033; 0.049 | <0.001 |
| *Hospital admission* | | | | | | | |
| General | 0.388 | 0.078; 0.698 | 0.014 | | 0.351 | 0.043; 0.659 | 0.026 |
| Technical | 0.395 | 0.140; 0.650 | 0.002 | | 0.373 | 0.120; 0.626 | 0.004 |
| Transport | 0.499 | 0.107; 0.891 | 0.013 | | 0.473 | 0.083; 0.863 | 0.018 |
| Administrative | 0.243 | -0.016; 0.502 | 0.065 | | 0.236 | -0.021; 0.493 | 0.071 |
| Care | 0.175 | -0.101; 0.451 | 0.213 | | 0.164 | -0.110; 0.438 | 0.240 |
| Agriculture | 0.06 | -0.259; 0.379 | 0.712 | | 0.058 | -0.261; 0.377 | 0.720 |
| Teaching | -0.024 | -0.406; 0.358 | 0.903 | | -0.041 | -0.421; 0.339 | 0.834 |
| Hospital admiss. |  |  |  | | 0.601 | 0.385; 0.817 | <0.001 |
| *Outpatient visits* | | | | | | | |
| General | 0.388 | 0.078; 0.698 | 0.014 | | 0.399 | 0.091; 0.707 | 0.011 |
| Technical | 0.395 | 0.140; 0.650 | 0.002 | | 0.395 | 0.142; 0.648 | 0.002 |
| Transport | 0.499 | 0.107; 0.891 | 0.013 | | 0.471 | 0.081; 0.861 | 0.018 |
| Administrative | 0.243 | -0.016; 0.502 | 0.065 | | 0.242 | -0.015; 0.499 | 0.065 |
| Care | 0.175 | -0.101; 0.451 | 0.213 | | 0.196 | -0.078; 0.470 | 0.162 |
| Agriculture | 0.06 | -0.259; 0.379 | 0.712 | | 0.093 | -0.226; 0.412 | 0.568 |
| Teaching | -0.024 | -0.406; 0.358 | 0.903 | | -0.025 | -0.405; 0.355 | 0.895 |
| Outpatient |  |  |  | | 0.373 | 0.244; 0.502 | <0.001 |
| *Contact family physician* | | | | | | | |
| General | 0.388 | 0.078; 0.698 | 0.014 | | 0.389 | 0.079; 0.699 | 0.014 |
| Technical | 0.395 | 0.140; 0.650 | 0.002 | | 0.398 | 0.143; 0.653 | 0.002 |
| Transport | 0.499 | 0.107; 0.891 | 0.013 | | 0.499 | 0.107; 0.891 | 0.013 |
| Administrative | 0.243 | -0.016; 0.502 | 0.065 | | 0.243 | -0.016; 0.502 | 0.066 |
| Care | 0.175 | -0.101; 0.451 | 0.213 | | 0.178 | -0.098; 0.454 | 0.206 |
| Agriculture | 0.060 | -0.259; 0.379 | 0.712 | | 0.061 | -0.258; 0.380 | 0.707 |
| Teaching | -0.024 | -0.406; 0.358 | 0.903 | | -0.022 | -0.404; 0.360 | 0.912 |
| Family Physic. |  |  |  | | 0.046 | -0.099; 0.191 | 0.534 |

^1^ Each domain is compared to the non-defined domains

^2^ Pooled data based on 15 imputations

* Two dummy variables, reference category is 0 sick days

** Quadratic term

Table C. Linear regression models of LRPD on occupational domain^1^, adjusted for age and work status (model 1), and additionally adjusted for ‘best’ health variables (models 2). Imputed data (n=2,531)^2^

|  | Model 1 | | | Model 2 | | |
| --- | --- | --- | --- | --- | --- | --- |
|  | Regression coefficient B | Confidence  Interval | Significance  (p-value) | Regression coefficient B | Confidence  Interval | Significance  (p-value) |
| General | 0.388 | 0.078; 0.698 | 0.014 | 0.130 | -0.170; 0.430 | 0.396 |
| Technical | 0.395 | 0.140; 0.650 | 0.002 | 0.205 | -0.040; 0.450 | 0.101 |
| Transport | 0.499 | 0.107; 0.891 | 0.013 | 0.290 | -0.086; 0.666 | 0.132 |
| Administrative | 0.243 | -0.016; 0.502 | 0.065 | 0.194 | -0.053; 0.441 | 0.124 |
| Care | 0.175 | -0.101; 0.451 | 0.213 | 0.039 | -0.226; 0.304 | 0.773 |
| Agriculture | 0.060 | -0.259; 0.379 | 0.712 | -0.082 | -0.392; 0.228 | 0.603 |
| Teaching | -0.024 | -0.406; 0.358 | 0.903 | 0.046 | -0.321; 0.413 | 0.804 |
| Obstructive lung disease |  |  |  | 0.360 | 0.162; 0.558 | <0.001 |
| Cardiovascular diseases |  |  |  | 0.325 | 0.178; 0.472 | <0.001 |
| Diabetes |  |  |  | 0.844 | 0.607; 1,081 | <0.001 |
| Cancer |  |  |  | 0.342 | 0.124; 0.560 | 0.002 |
| Activity limitations (1-3) |  |  |  | 0.239 | 0.131; 0.347 | <0.001 |
| Self-rated health (1-5) |  |  |  | -0.037 | -0.117; 0.043 | 0.370 |
| Cognitive functioning (0-30) |  |  |  | -0.091 | -0.116; -0.066 | <0.001 |

^1^ Each domain is compared to the non-defined domains

^2^ Pooled data based on 15 imputations


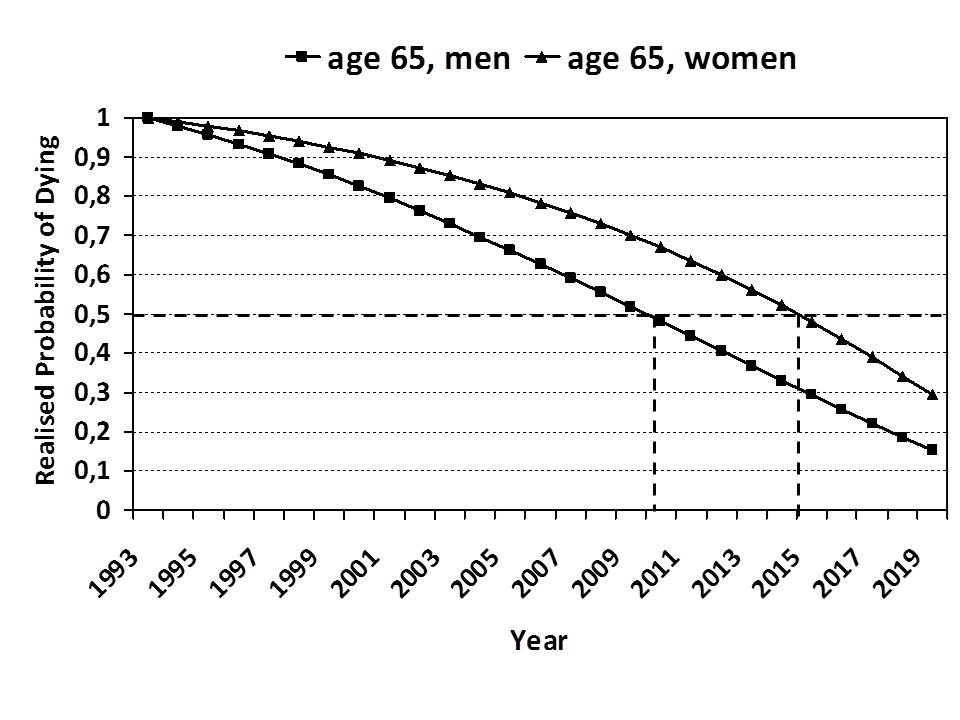


Figure A. Sex-specific survival curves at age 65, derived from the Realized Probability of Dying (RPD). The horizontal dashed line indicates the median RPD. The points where this line crosses the survival curves indicate the average life expectancies of men and women at age 65; these life expectancies can be read on the x-axis by following the vertical dashed lines and subtracting 1993. Likewise, the point where a horizontal line starting from any point on the y-axis crosses a survival curve, indicates how an RPD-value translates into the corresponding life expectancy at age 65.
